# PTIP UFMylation promotes replication fork degradation in BRCA1-deficient cells

**DOI:** 10.1016/j.jbc.2024.107312

**Published:** 2024-04-22

**Authors:** Qunsong Tan, Xingzhi Xu

**Affiliations:** 1 Guangdong Key Laboratory for Genome Stability & Disease Prevention and Carson International Cancer Center, Marshall Laboratory of Biomedical Engineering, Shenzhen University Medical School, Shenzhen, China; 2 Guangdong Key Laboratory for Biomedical Measurements and Ultrasound Imaging, National-Regional Key Technology Engineering Laboratory for Medical Ultrasound, School of Biomedical Engineering, Shenzhen University Medical School, Shenzhen, China

**Keywords:** UFMylation, PTIP, BRCA1, replication fork, end resection

## Abstract

Homologous-recombination deficiency due to breast cancer 1/2 (BRCA1/2) mutations or mimicking BRCA1/2 mutations confer synthetic lethality with poly-(ADP)-ribose polymerase 1/2 inhibitors. The chromatin regulator Pax2 transactivation domain interacting protein (PTIP) promotes stalled replication fork degradation in BRCA1-deficient cells, but the underlying mechanism by which PTIP regulates stalled replication fork stability is unclear. Here, we performed a series of *in vitro* analyses to dissect the function of UFMylation in regulating fork stabilization in BRCA1-deficient cells. By denaturing co-immunoprecipitation, we first found that replication stress can induce PTIP UFMylation. Interestingly, this post-translational modification promotes end resection and degradation of nascent DNA at stalled replication forks in BRCA1-deficient cells. By cell viability assay, we found that PTIP-depleted and UFL1-depleted BRCA1 knockdown cells are less sensitive to poly-(ADP)-ribose polymerase inhibitors than the siRNA targeting negative control BRCA1-deficient cells. These results identify a new mechanism by which PTIP UFMylation confers chemoresistance in BRCA1-deficient cells.

Homologous recombination (HR)-mediated repair of replication-induced double-strand breaks (DSBs) and stabilization of stalled replication forks is essential for ensuring genome stability. As such, deficiencies in HR can cause chemoresistance, in particular due to synthetic lethality to topoisomerase or poly-(ADP)-ribose polymerase inhibitors (PARPi) (1, 2, 3, 4). Such HR-deficient cells have been recently defined as exhibiting “BRCAness” specifically because cells or tumors harboring breast cancer 1 (BRCA1) or BRCA2 mutations are unable to repair DSBs *via* HR (5). These cells with an HR deficiency carry deficiencies in HR-related or DNA damage signaling genes, including RAD51, RPA1, ATR, ATM, and FANCA (6). Many DNA damage response (DDR) proteins, including BRCA1 or BRCA2, can regulate stalled replication fork degradation or protection. Known DDR factors that ensure replication fork protection and confer PARPi-resistance in BRCA1-deficient cells are, however, elusive. The identification of such factors that confer PARPi chemoresistance to BRCA1-deficient cells will promotes pharmacological effects on patients with BRCA1/2 mutations and favor patient outcome.

The six BRCT domains-containing protein PTIP (Pax2 transactivation domain interacting protein) involves DNA DSBs repair pathways and is essential for embryogenesis (7, 8). PTIP associates with MLL2-3 complex and may regulate gene expression through promoting methylation of H3K4 (9). In addition, PTIP accumulates at DSBs to promotes class recombination switch and genome stability, which is independent on its interaction with MLL3-4 complex (10). Further, mutation of PTIP binding site in 53BP1(S25A) allows DNA2-dependent hyper-resection, indicating that PTIP may suppress DNA end resection through DNA2 pathway (11). On the other hand, PTIP promotes stalled replication fork degradation in mouse BRCA-deficient B lymphocytes cells, and loss of PTIP causes chemoresistance to PARP inhibitors (olaparib, rucaparib, and niraparib) or cisplatin (4).

A plethora of studies have identified that post-translational modifications, including ubiquitination, sumoylation, phosphorylation, and poly-(ADP-ribosyl)ation, are important for regulating end resection at stalled replication forks (12, 13, 14). UFMylation is one of the most recently identified post-translational modifications involved in this process. Much like ubiquination, UFMylation is catalyzed sequentially by an UFM1-activating enzyme (E1, UBA5), UFM1-conjugating enzyme (E2, UFC1), and UFM1-ligase (E3, UFL1), the only known E3 ligase so far.

Since its discovery, UFMylation has since been extensively studied in the context of DDR and ER-phagy (15, 16, 17). In our previous work, we determined the involvement of UFMyaltion in DNA DSBs repair pathway (15). To further explore its multifunction, we utilized both immunoprecipitation–mass spectrometry and denaturing co-immunoprecipitation (co-IP) to screen for the UFMylation of DDR factors involved specifically in DNA end resection at DSBs or stalled replication forks. In this study, we aimed to characterize the role of UFMylation of PTIP in replication fork degradation in BRCA1-deficient cells. In brief, we showed that PTIP was UFM1-modified at K148, K173, K175, and K176, allowing PTIP to promote DNA end resection and nascent DNA degradation at stalled replication forks in BRCA1-deficient cells. Interestingly, loss of UFL1 in BRCA1-deficient cells resulted in chemoresistance to PARPi. Thus, PTIP UFMylation seems essential to eliciting chemoresistance of BRCA1-deficient cells to PARPi by regulating stalled replication fork stability.

## Results

### PTIP is UFMylated *in vivo* in response to replication stress

**Figure 1:**
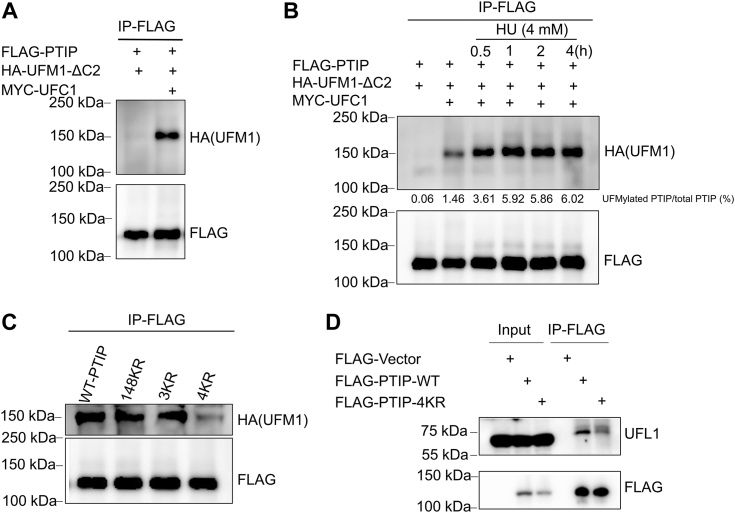
**PTIP is UFMylated *in vivo* in response to replication stress.***A*, FLAG-PTIP and HA-UFM1-ΔC2 were cotransfected into HEK293T cells with or without MYC-UFC1 (E2). Cells were subjected to FLAG immunoprecipitation under denaturing conditions and analyzed by Western blotting using an anti-HA or anti-FLAG antibody. *B*, HEK293T cells cotransfected with FLAG-PTIP, HA-UFM1-ΔC2, and MYC-UFC1 were treated with HU (4 mM) and collected at the indicated time points. FLAG-PTIP proteins were pulled down by FLAG-M2 beads, and PTIP UFMylation was examined by Western blotting with an anti-HA or anti-FLAG antibody. *C*, wildtype (WT) and mutant PTIP constructs were transfected with HA-UFM1-ΔC2 and MYC-UFC1 into HEK293T cells, and UFM1-conjugated PTIP was detected by Western blotting. *D*, HEK293T cells transfected with FLAG-PTIP^WT^ and FLAG-PTIP^4KR^ were collected for immunoprecipitation followed by Western blotting. HU, hydroxyurea; PTIP, PAX interacting protein 1.

In an earlier work, we leveraged immunoprecipitation-mass spectrometry to screen for UFM1-modified substrates involved in the DDR (15). As pro-form UFM1 is processed by protease UfSP1/2 into active form UFM1-ΔC2 (last two amino acids at the C terminal of UFM1 were depleted), we optimized the UFMylation assay system *in vivo*, which only included plasmids of substrate, HA-UFM1-ΔC2, and MYC-UFC1 (E2). We identified PTIP as one UFM1 target, but the function of PTIP UFMylation was unknown. Here, we first confirmed that PTIP is UFM1-modified. To do so, we cotransfected FLAG-tagged PTIP and HA-UFM1-ΔC2 (active form of UFM1), with or without MYC-tagged UFC1, into 293T cells. Under denaturing conditions, we were able to pull down HA-UFM1-ΔC2-conjugated FLAG-PTIP with FLAG-M2 beads. Western blot analysis showed an obvious HA band (∼150 kD) higher than the FLAG-PTIP band (<150 kD) only in the E2 MYC-UFC1 cotransfection group (Fig. 1*A*), indicating that this shift in the HA-band corresponds to UFM1-modified PTIP in cells.

To explore the function of PTIP UFMylation, we queried whether replication stress (induced by hydroxyurea [HU]) regulates this UFMylation event. To this aim, we repeated the cotransfection detail above, but this time treated the cells with HU. Denaturing co-IP showed that the PTIP UFMylation level increased at different time points to 2 h (Fig. 1*B*) and then remained stable from 2 to 4 h after HU treatment (Fig. 1*B*).

Next, we wanted to identify the PTIP UFMylation site(s). To do so, we constructed various PTIP (1069 amino acids) fragments. We transfected these PTIP fragments individually with our UFMylation assay plasmids into HEK293T cells and performed denaturing co-IP followed by Western blotting which indicated that the PTIP 170 to 205 amino acids were essential for PTIP UFMylation (Fig. S1, *A* and *B*). This region contains three lysine (K) residues. We thus constructed various point mutants, converting K173, K175, and K176 to K173R, K175R, K176R, K173/175R, K173/176R, K175/176R, or K173/175/176R (3KR); here, we found that the triple mutation markedly decreased PTIP UFMylation (Fig. S1*B*). During the process of PTIP UFMylation site (s) screening, we found that K148R mutant also reduced PTIP UFMylation level when compared with WT-PTIP UFMylation (Fig. 1*C*). Then, we generated K148/173/175/176R (4KR) mutants. When we compared the UFMylation levels of these two PTIP mutans from long exposure development (Fig. 1*C*), we observed that UFMylation of 4KR mutant was completely inhibited. Thus, we concluded that based on 4KR mutant UFMylation level markedly lower than K148R or K173/175/176R mutant, K148, K173, K175, and K176 (4K) were the PTIP UFMylation sites. At the same time, we found that 4KR mutant did not interact with the E3 ligase UFL1 (Fig. 1*D*).

To investigate whether UFMylation regulates PTIP recruitment to DSBs, we performed a micro-irradiation (IR) laser assay both in HeLa and U2OS cells. The PTIP-4KR mutant was still recruited to DSBs to the same degree following micro-IR when compared with WT-PTIP in both kinds of cells (Fig. S1*C*). We next explored the role of PTIP UFMylation in regulating DSB end resection in HeLa cells. Previous report shows that RPA32 S33 phosphorylation (pRPA32-S33) serves as a marker for DNA end resection (18), thus we examined whether PTIP UFMylation regulated pRPA32-S33 after IR treatment. We found that pRPA32-S33 levels in PTIP-depleted HeLa cells were much higher than that in the control group cells after IR treatment and rescued by re-expression of wildtype PTIP, but not UFMylation-defective mutant PTIP-4KR (Fig. S1*D*), indicating that UFMylation of PTIP does not regulate its inhibitory role on DSB end resection. We also found that the PTIP UFMylation levels were slightly increased in S/G2/M phase when compared with G1 phase (Fig. S1*E*). Altogether, these data suggest that replication stress induces PTIP UFMylation, which might in turn be involved in regulating stalled replication forks.

### PTIP UFMylation promotes end resection following replication stress

**Figure 2:**
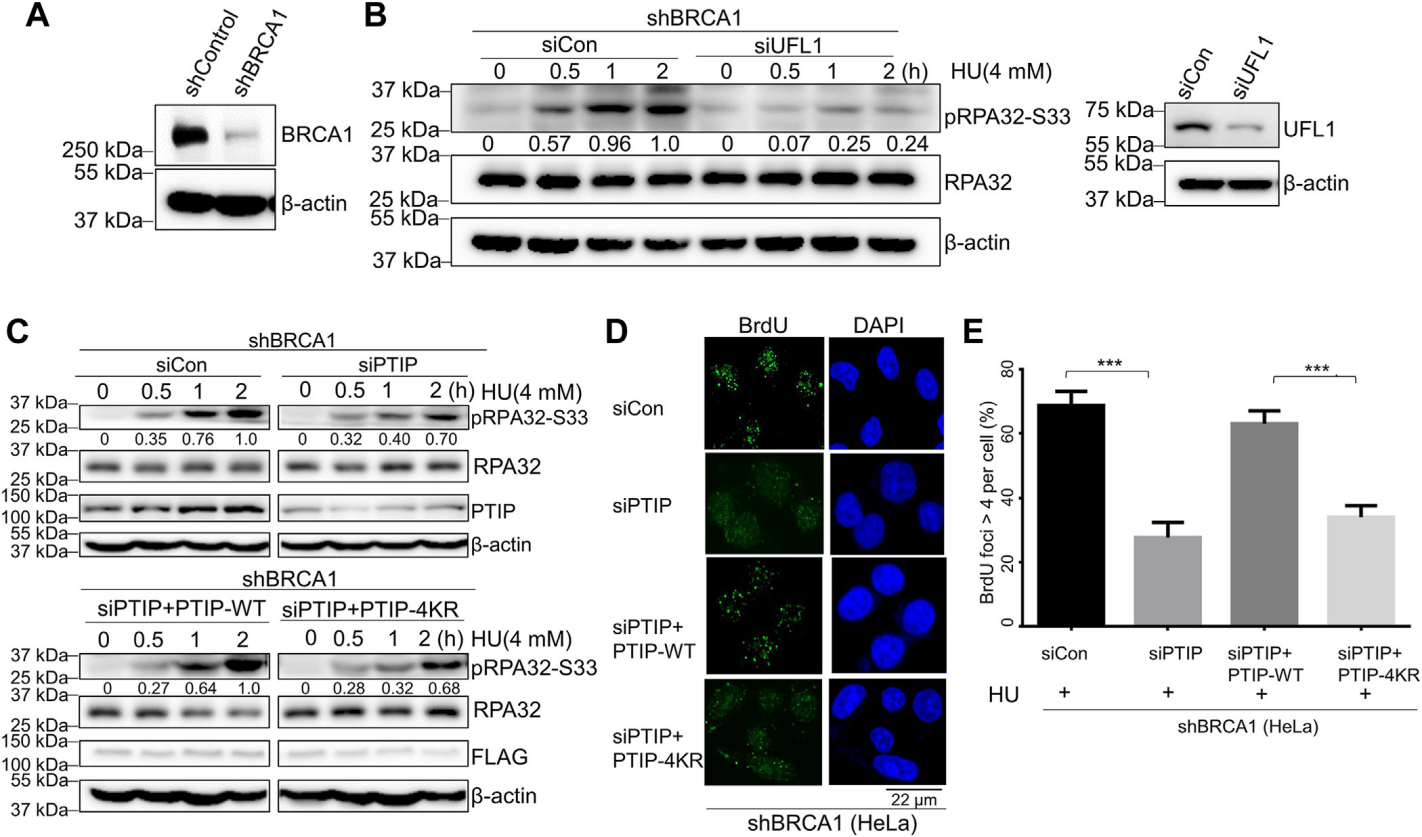
**UFMylation promotes end resection following replication stress.***A*, shControl HeLa and shBRCA1 HeLa cells were harvested for Western blotting to check the BRCA1 knockdown efficiency. *B*, shBRCA1 HeLa cells transfected with siUFL1 were treated with HU (4 mM) and harvested at the indicated time points for Western blotting with anti-RPA32-S33, β-actin, and UFL1 antibodies. *C*, shBRCA1 HeLa cell lines transfected with siPTIP were reconstituted with FLAG-PTIP^WT^ and FLAG-PTIP^4KR^, treated with HU (4 mM), and harvested at the indicated time points for Western blotting. *D* and *E*, PTIP-depleted shBRCA1 HeLa cells were reconstituted with FLAG-PTIP^WT^ and FLAG-PTIP^4KR^ and harvested for native BrdU assay. Quantification of the number of BrdU foci >4 per cell is shown (*E*). The data represent the means ± SD (n = 3 biological replicates, ∗∗∗*p* ≤ 0.001). BRCA1, breast cancer 1; BrdU, bromo-deoxyuridine; HU, hydroxyurea; PTIP, PAX interacting protein 1.

Based on our results thus far, we considered that UFMylation might regulate PTIP function at stalled replication forks. Indeed, PTIP promotes replication fork degradation in both BRCA1- and BRCA2-deficient cells (19). We therefore constructed BRCA1-deficient HeLa cells using a lentivirus-mediated shRNA targeting BRCA1. Upon examining the BRCA1 protein levels in our shBRCA1 cell line, we saw that BRCA1 levels were decreased by ∼90% compared to shControl cells (Fig. 2*A*).

Once we had generated our cellular model system, we could test whether UFMylation might regulate end resection at stalled replication forks. We first examined the effects of UFL1 depletion in our shBRCA1 HeLa cells, focusing on pRPA32-S33 after HU-induced replication stress. siRNA-mediated UFL1 depletion markedly decreased the pRPA32-S33 levels when compared with that in control shBRCA1 HeLa cells at 1 and 2 h after HU treatment (Fig. 2*B*), indicating that UFL1 directly promotes end resection in BRCA1-deficient cells upon replication stress.

To confirm our previous hypothesis, we used siRNA to knock down PTIP in our shBRCA1 cell line, and consistent with our previous results, we found that pRPA32-S33 levels were further decreased compared to siCon shBRCA1 HeLa cell at 1 and 2 h post HU treatment (Fig. 2*C*). We thus conclude that PTIP promotes end resection and 3′ prime ssDNA formation.

To further investigate this possible role for PTIP UFMylation, we transfected siRNA-resistant plasmids separately expressing FLAG-WT-PTIP or FLAG-PTIP-4KR mutant into PTIP-depleted shBRCA1 HeLa cells. All cells were treated with HU (4 mM) and collected at indicated timepoints for Western blotting. We observed lower pRPA32-S33 protein levels in the PTIP-4KR mutant expression group cells upon 1 or 2 h post HU treatment compared with that in PTIP-WT expression group shBRCA1 cells (Fig. 2*C*). We repeated the experimental set up but used ssDNA immunofluorescent assays to examine the role of PTIP UFMylation of PTIP in ssDNA formation during HU treatment. Consistent with our earlier findings, we saw that the number of bromo-deoxyuridine (BrdU) foci, directly representing ssDNA, in our siCon group was about 40% more than that in the siRNA-mediated PTIP-depletion group (Fig. 2, *D* and *E*). Moreover, we saw about 30% less BrdU foci accumulation in PTIP-4KR mutant cells compared with that in PTIP-WT shBRCA1 cells (Fig. 2, *D* and *E*). Collectively, these results support that UFL1-mediated PTIP UFMylation promotes ssDNA formation after HU-mediated stalling of replication forks in BRCA1-deficent cells.

### PTIP UFMylation promotes nascent DNA degradation in BRCA1-deficient cells

**Figure 3:**
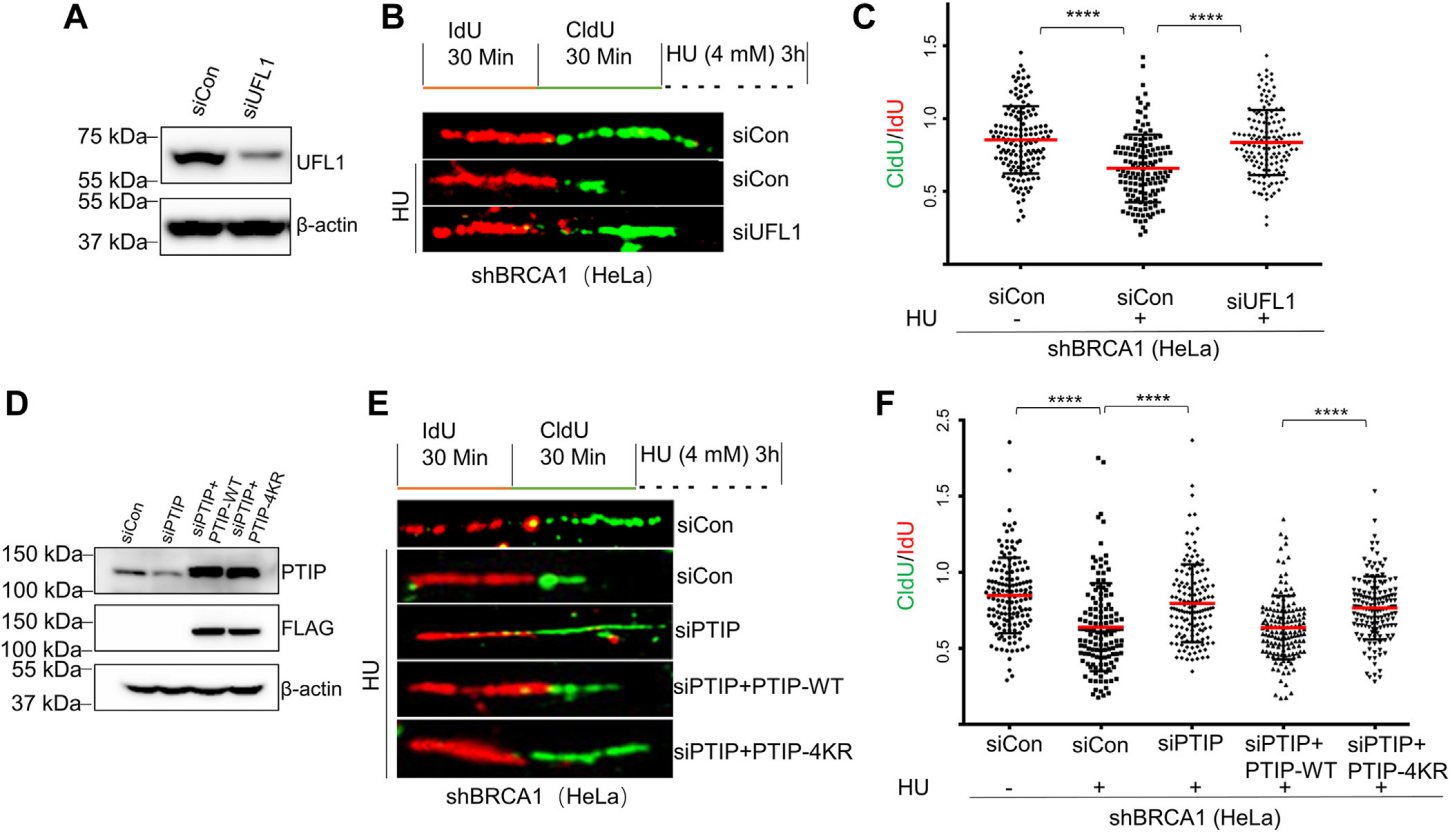
**UFMylation promotes nascent DNA degradation in BRCA1-deficient cells.***A*–*C*, shBRCA1 HeLa cells were transfected with the indicated siRNAs for 48 h (A), then all cells were sequential labeled with IdU (50 μM) and CldU (100 μM) for 30 min followed with or without HU (4 mM, 3 h) exposure, and prepared for DNA fiber assay (*B*). At least 150 events were quantified for each condition (*C*). *D–F*, shBRCA1 HeLa cells expressing siPTIP or control (siCon) were complemented with FLAG-PTIP^WT^ and FLAG-PTIP^4KR^ (*D*). DNA fiber assays were performed (*E*), and at least 150 events were quantified in each condition (*F*). At least three experiments were performed (∗∗∗∗*p* ≤ 0.0001). BRCA1, breast cancer 1; CldU, chloro-deoxyuridine; HU, hydroxyurea; IdU, iodo-deoxyuridine; PTIP, PAX interacting protein 1.

In our next set of analyses, we evaluated stalled replication fork stability in BRCA1-deficient cells, using a DNA fiber assay (20). As UFL1 promotes end resection at stalled replication forks, we treated shBRCA1 HeLa cells with HU to stall the replication forks and induce their reversal, and then examined nascent DNA degradation. We depleted UFL1 by siRNA in the BRCA1-deficient cells (Fig. 3*A*) and then sequentially labeled them with iodo-deoxyuridine (IdU) (red) followed by chloro-deoxyuridine (CldU) (green), before exposing them to HU. By analyzing the length of IdU and CldU fiber, we observed that the CldU/IdU tract ratio was 0.62 in siCon shBRCA1 cells, while the same ratio in the UFL1-depleted shBRCA1 HeLa cells with HU treatment was 0.85; these values indicate that UFL1 depletion abolishes fork degradation in shBRCA1 cells in response to HU treatment (Fig. 3, *B* and *C*).

Depletion of PTIP in mouse B cells prevents fork degradation. To confirm that it is the UFMylation of PTIP that regulates fork degradation in BRCA1-deficient cells, we knocked down PTIP by siRNA in shBRCA1 cells (Fig. 3*D*). We observed that CldU/IdU tract ratio was 0.64 in siCon shBRCA1 cells after HU treatment, while the same ratio in PTIP-depleted group was 0.79, indicating that PTIP promotes fork degradation in shBRCA1 HeLa cells. We also transfected siRNA-resistant plasmids expressing PTIP-WT or PTIP-4KR mutant into PTIP-depleted shBRCA1 cells (Fig. 3*D*) and performed a DNA fiber assay. Consistent with our previous results, we found that PTIP-WT can rescue the fork degradation upon HU treatment, while overexpression of the PTIP-4KR mutant prevented fork degradation in PTIP- and BRCA1-depleted cells (Fig. 3, *E* and *F*). To explore whether PTIP directly regulates nucleases at replication fork, we screened the interaction between PTIP and nucleases which mainly functions in replication fork protection. By performing co-IP, we found that PTIP did not interact with MRE11 (Fig. S2*A*), which was thought the main regulator in degrading stalled replication fork. Interestingly, we observed that PTIP interacted with CtIP and SLX4 (Fig. S2, *B* and *C*). To investigate whether PTIP UFMylation regulates recruitment of MRE11 or CtIP at replication fork, we performed proximity ligation assay (PLA) in BRCA1-depleted HeLa cells with or without HU treatment and found that inhibition of PTIP expression reduced the HU treatment-induced increase of both MRE11/EdU PLA foci (Fig. S2*D*) and CtIP/EdU PLA foci (Fig. S2*E*), whereas re-expression of wildtype PTIP, but not UFMylation-defective mutant PTIP-4KR, rescued HU treat-induced increase of both PLA foci (Fig. S2, *D* and *E*), indicating PTIP UFMylation promotes both MRE11 and CtIP recruitment to the stalled replication fork. These results demonstrate that UFL1-mediated UFMylation of PTIP is essential for stalled fork degradation in BRCA1-deficient cells.

### Loss of UFL1 confers chemoresistance to PARPi in BRCA1-deficient cells

**Figure 4:**
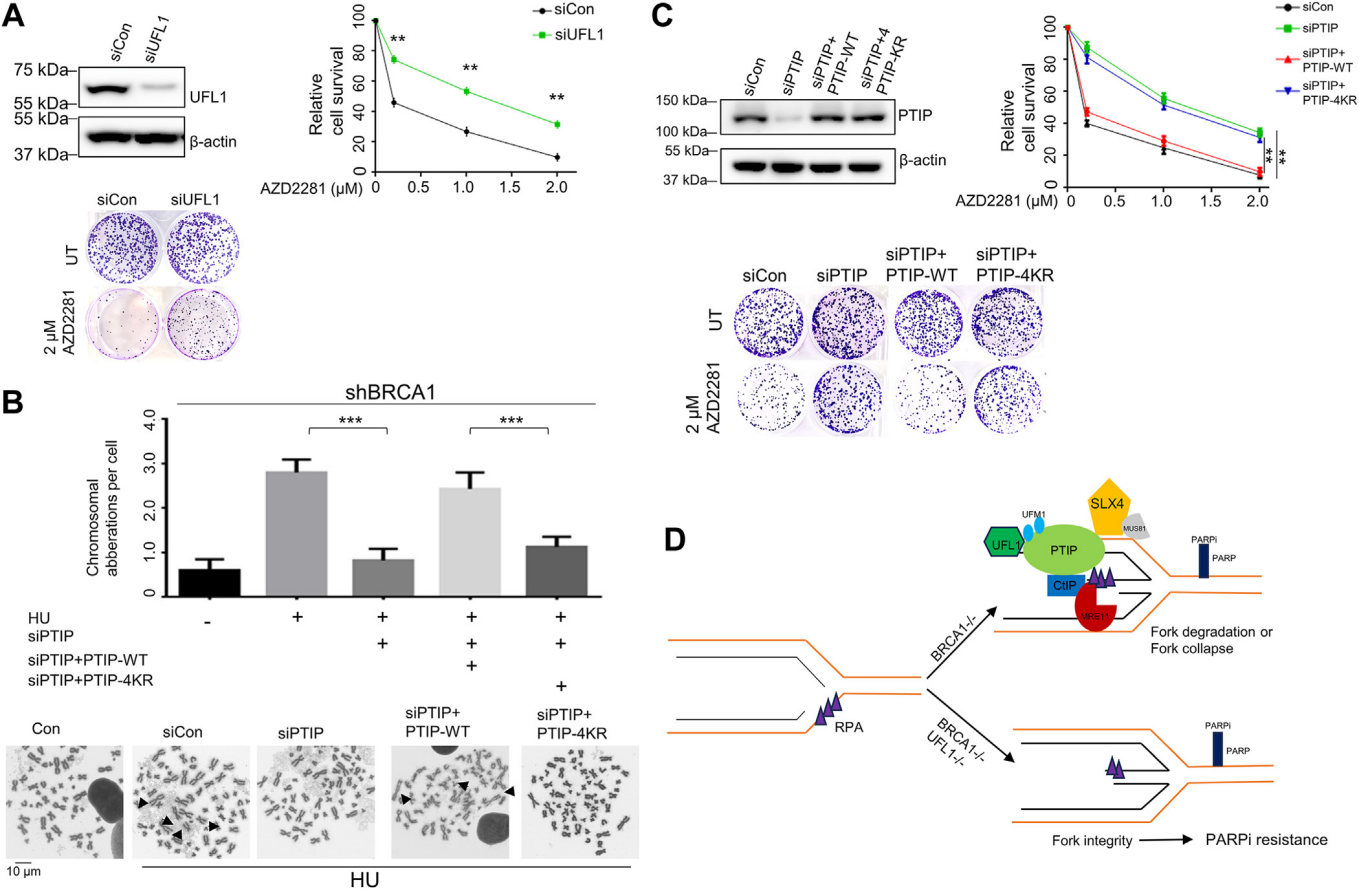
**Loss of UFL1 confers BRCA1-deficient cells chemoresistance to PARP inhibitor.***A*, clonogenic assay (*top right*) and representative images (*bottom*) of shBRCA1 HeLa cells expressing negative control (siCon) siRNA or siUFL1 and exposed to increasing concentrations of PARPi (Olaparib). Western blot analysis (*top left*) of cells expressing the siCon or siUFL1 at 72 h following transfection. *B*, shBRCA1 HeLa cells were transfected with the indicated siRNAs and then reconstituted with FLAG-PTIP^WT^ and FLAG-PTIP^4KR^. Chromosome breakages were analyzed (*upper*) and representative images are shown (*lower*). *C*, clonogenic assay (*top right*), representative images (*bottom*) and Western blot analysis (*top left*) of shBRCA1 HeLa cells transfected with the indicated siRNAs and complemented with FLAG-PTIP^WT^ and FLAG-PTIP^4KR^ and then exposed or not to increasing concentrations of PARPi (Olaparib). The data represent the means ±SD (∗∗*p* ≤ 0.01; ∗∗∗*p* < 0.001.). *D*, model for loss of UFL1-mdiated PTIP UFMylation confers PARPi resistance in BRCA1-deficient cells. UFL UFMylates PTIP at K148/173/175/176 and promotes recruitment of PTIP at stalled replication fork. Then, UFMylated PTIP allows binding of CtIP or SLX4, ultimately promoting fork degradation or collapse. As UFL1 is depleted, PTIP is not UFMylated, and CtIP or SLX4 recruitment to stalled replication fork is reduced, resulting fork stabilization. Low UFL1 expression level causes PARPi resistance in BRCA1-deficient cells. BRCA1, breast cancer 1; PARPi, poly-(ADP)-ribose polymerase inhibitors; PTIP, PAX interacting protein 1.

HR-deficient cancers are typically treated with platinum and PARP inhibitors (6). Targeting BRCA mutant cells with PARPi results in cell cycle arrest and apoptosis, such that BRCA-deficient tumors are selectively killed (2). Replication fork protection, however, induces chemoresistance in HR-deficient cancer cells (19, 21). Thus far, we have seen that PTIP UFMylation promotes replication fork degradation in BRCA1-deficient cells, implying that knocking down UFL1 or PTIP-4KR mutant stabilizes stalled replication forks and confers resistance to PARPi in this context. We thus finally established whether UFMylation regulated PARPi resistance in BRCA1-deficient HeLa cells. To do so, we examined the viability of shBRCA1 HeLa cells after depleting UFL1. We found that siRNA-mediated UFL1 depletion BRCA1-deficient cells were less sensitive to PARPi than siCon shBRCA1 group cells (Fig. 4*A*).

To confirm a role for UFMylation in chemoresistance in BRCA1-deficient cells, we examined the effects of PTIP UFMylation on chromosome aberrations. We found that both depletion of PTIP and overexpression of a PTIP-4KR mutant could reduce the number of chromosomal aberrations in BRCA1-deficient cells when compared with siCon or PTIP-WT shBRCA1 group cells (Fig. 4*B*). Cell viability demonstrated that loss of PTIP in BRCA1-deficient cells or expression of a PTIP-4KR mutant in PTIP- and BRCA1-depleted cells showed chemoresistance to PARPi, while expression of PTIP-WT in PTIP- and BRCA1-depleted cells rescued the hypersensitivity to PARPi (Fig. 4*C*).

Taken together, we propose a working model (Fig. 4*D*) by which UFL1 UFMylates PTIP in response to replication stress (here, HU exposure) and promotes PTIP accumulation at stalled replication forks in BRCA1-deficient cells. Then, PTIP promotes the recruitment of nucleases to the stalled replication forks, which degrade or resolve the stalled replication fork in BRCA1-deficient cells. Finally, loss of UFL1 confers HR-deficient cells resistant to PARPi. As such, UFL1 can be considered a biomarker of BRCA1-deficient cancer cells and thus indicate the likely response to chemotherapy.

## Discussion

In this study, we aimed to understand the function of PTIP UFMylation in PARPi resistance in BRCA1-deficient cells. Through a series of *in vitro* and *in vivo* analyses, our study uncovered a new role of PTIP UFMylation in promoting stalled replication fork degradation in BRCA1-deficient cells in response to replication stress. We first showed that PTIP was modified by UFM at K148, K173, K175, and K176 *in vivo* (Fig. 1*C*). This modification was induced by HU treatment—induced replication stress (Fig. 1*C*), while did not exhibit a significant role in PTIP recruitment to DSBs or inhibition of DSB end resection (Fig. S1, *C* and *D*); rather, our data indicate that UFMylation helps regulate stalled replication forks through nascent DNA degradation. Further, PTIP promotes the recruitment of nucleases to the stalled replication forks, which degrade or resolve the stalled replication fork in BRCA1-deficient cells (Fig. 4*D*).

Previous studies have shown that loss of PTIP leads to replication fork protection in BRCA1/2-deficient mouse cells (19). We have built on these findings by showing that UFL1-mediated PTIP UFMylation promotes ssDNA formation (Fig. 2, *C* and *D*) and nascent DNA degradation upon HU treatment in BRCA1-deficient cells (Fig. 3, *E* and *F*). Results from other recent studies suggest that replication fork protection correlates with resistance to PARPi (21). We found that both depletion of UFL1 and PTIP caused chemoresistance of BRCA1-deficient cells to PARPi by stabilizing stalled replication forks (Fig. 4, *A* and *C*). We therefore propose that loss of UFL1 confers drug resistance to PARPi due to lost recruitment of PTIP to stalled replication forks (Fig. 4*D*).

Through this work, we have uncovered a new role for PTIP UFMylation in regulating replication fork stability; previous studies showed that MRE11 regulated replication fork stability (3, 19) and multiple mechanisms of PARPi resistance (22); however, the specific mechanism of how PTIP UFMylation by UFL1 regulates MRE11 recruitment to stalled replication forks remains unknown, which will provide evidence for development and application of UFMylation inhibitors for targeted therapy. The phosphorylation of CtIP by ATM or CDK2 is required to stimulate MRE11 nuclease activity in DNA end resection (23, 24, 25). The results of previous studies showed that neither PTIP nor MLL2/3 interacts with MRE11 *in vivo* (26). We confirmed that PTIP did not interact with MRE11 (Fig. S2*A*), while found that PTIP interacted with CtIP (Fig. S2*B*). Furthermore, PTIP UFMylation promoted recruitment of both CtIP and MRE11 to the stalled replication forks in BRCA1-deficient cells (Fig. S2, *D* and *E*). Therefore, we speculate that PTIP UFMylation indirectly promotes MRE11 recruitment to the replication stress-induced stalled forks in BRCA1-deficient cells, possibly facilitated by CtIP.

The PTIP protein contains six BRCT (BRCA1 C-terminal domain) domains that are implicated in many DNA damage pathways (7). BRCT domains are important modules to transduce target protein signaling *via* the binding of phosphorylated sites (27). We posit that PTIP binds to and stabilizes CtIP at stalled replication forks, which then cooperates with MRE11 in DNA end resection. Indeed, we found that PTIP interacts with CtIP *in vivo* (Fig. S2*B*). Data from studies investigating the function of Rtt107, the budding yeast homologue of PTIP, indicate that the Rtt107 BRCT3/4 and 5/6 domains are essential for the recruitment of scaffold protein Slx4 to DNA lesions in yeast, implicating that PTIP might also interact with SLX4 in human cells (28, 29, 30). Indeed, we have seen that PTIP can indeed interact with SLX4 in human cells (Fig. S2*C*). As human SLX4 recruits nuclease MUS81 and SLX1 to resolve recombination intermediates (31, 32), future studies into how the UFMylation of PTIP affects the SLX4-MUS81 complex could reveal the mechanism of degradation or resolution of stalled replication fork in BRCA1-deficient cells.

## Experimental procedures

### Cell lines

HEK293T and HeLa cells were cultured in high-glucose Dulbecco's modified Eagle's medium containing 10% FBS and incubated at 37 °C in a humidified 5% CO_2_ incubator.

A lentivirus was used to knockdown BRCA1 in HeLa cells with the following shRNA cloned into a pLKO.1 vector: sense: 5′-ATTCATGCCAGAGGTCTTATA. As previously described (13), the lentiviral particles were produced by transfecting both pLKO.1-shRNA and packaging plasmids in 293T cells. Then, HeLa cells were infected with the lentiviral particles and selected using puromycin. BRAC1 depletion was confirmed by Western blotting.

### Plasmid and transfection

PTIP fragment was produced by amplification from 293T cDNA and were subcloned into a pEGFP-C1B or pcDNA3.1 FLAG vector using a seamless cloning kit (TransGen). PTIP mutant plasmids were amplificated by PCR with corresponding primers and digested with DpnI for transformation. All plasmid sequences were confirmed by sequencing. HA-UFM1-Δ2, His-UFL1, and His-UFC1(E2) plasmids were kindly provided by Dr Gong Yamin in our lab. Plasmids or siRNAs were transfected into HeLa cells with PEI reagent or Lipofectamine3000 according to the manufacturer’s protocol.

siRNAs were transfected using Lipofectamine RNAiMAX (Invitrogen), as per the manufacturer’s protocol, to knockdown PTIP or UFL1. The following siRNAs were used:

PTIP-1 sense: AAGGAAGAAGAGGAAGAGGAA.

PTIP-2 sense: ACACTGAGGAATATTACTA.

UFL1-3′UTR sense: GAAACACTTCTGTGTCAGAAA.

### Immunoprecipitation

Flag- or HA-tagged proteins were transiently expressed in 293T or HeLa cells. For natural immunoprecipitation, cells were washed with cold PBS twice and lysed with IP buffer containing 50 mM tris-HCl (pH 7.5), 150 mM NaCl, 0.5% NP-40, 1 mM EDTA, phosphatase inhibitors, and proteinase inhibitors. Cell lysates were pelleted by centrifugation at 12,000*g* and 4 °C for 30 min, followed by centrifugation at 4 °C and 12,000*g* for 10 min. For denaturing immunoprecipitation, cells were washed twice with cold PBS and lysed in 5xSDS buffer containing 250 mM Tris-HCl (pH 7.5), 500 mM NaCl, and 5% SDS, and boiled at 100 °C for 30 min. Then, cell lysates were digested with benzonase and diluted 5-fold in RIPA buffer. The diluted lysates or natural lysates were centrifuged at 12,000*g* at 4 °C for 15 min, and the FLAG-tagged proteins were purified from the soluble lysis by adding anti-FLAG (M2) beads. After rotating overnight at 4 °C, the M2 beads were washed with IP buffer at least three times and boiled with SDS loading buffer at 100 °C for 10 min.

### Western blotting

Western blotting was performed as previously described (15). The primary antibodies were used at the following dilutions: anti-PTIP (1:1000, Bethyl), anti-CtIP (1:1000, Proteintech), anti-CtIP (1:500, Santa Cruz), anti-RPA32-S33 (1:1000, Bethyl), anti-β-actin (1:3000, Abclonal), anti-FALG (1:2000, Invitrogen), anti-HA (1: 1000, Proteintech), anti-UFL1 (1:1000, Bethyl), anti-GST (1:1000, Abclonal), and anti-His (1:1000, Abclonal).

### Native BrdU immunofluorescence

For native BrdU immunofluorescence staining, HeLa cells were seeded on 18 mm × 18 mm glass coverslips. BrdU (20 μM) was incorporated into cells for 36 h, and HU (4 mM) was added into cells during the last 2 h BrdU treatment. Cells were pre-extracted and fixed as previously described. Briefly, cells were pre-extracted with PE buffer containing 0.1% Triton-X100 for 5 min and fixed with PFA for 15 min at room temperature. Then, cells were treated with 0.2% Triton-X100 in PBS for 10 min, washed three times with PBST, and blocked with 5% BSA in PBS for 1 h. Then, the cells were incubated with anti-BrdU (1:500, Abcam) for 1 h at room temperature, washed three times with PBST, and incubated with fluorescence secondary antibodies for 1 h, at room temperature in the dark. Finally, the cells were washed and stained with DAPI for 2 min. After washing with ddH_2_O, the coverslips were mounted on slides with anti-fade (Abcam). Images were captured at 63× magnification using an Andor Dragonfly system. Quantification of nuclear foci was performed using ImageJ.

### DNA fiber assay

DNA fiber assay was performed as previously described (19). In brief, cells were labeled sequentially with IdU (50 μM) and CldU (100 μM) for the indicated times. To test replication fork stability, HU (4 mM) was added after CldU labeling for 3 h. Then, the cells were harvested and resuspended in cold PBS at a density of 100, 000 cells/ml. Draw line on a glass slide with 2 μl of cell suspension to allow dry to tackiness, and 15 μl lysis buffer [200 mM Tris-HCl (pH7.4), 50 mM EDTA, 0.5% SDS] was added into the cell lines. After 10 min, the slides were tilted at a 25° angle to allow the DNA fibers to run to the bottom of the slide. DNA spreads were air dried and fixed in 3:1 methanol: acetic acid for 2 min. The fibers were treated with freshly prepared 2.5 M HCl for 60 min, washed with PBST (PBS+0.1% Tween), and blocked with 5% BSA in PBST for 30 min. The fibers were incubated with anti-BrdU antibodies [IdU (1:250, B44, 347580; BD); CldU (1:250, ab6326; Abcam)] for 2 h at room temperature. Anti-mouse Alexa 546 (1:250; Molecular Probes, A21123) and anti-rat Alexa 488 (1:250; Molecular Probes, A21470) secondary antibodies were incubated on the slides for 2 h at room temperature in the dark. After washing three times with PBST, the slides were air dried and mounted with Prolong Gold Antifade (P30930; ThermoFisher). Images of the fibers were acquired under a 60 × /1.4 oil immersion objective (Nikon Eclipse Ti2 and Andor Fusion software).

### Micro-IR assay

HeLa cells or U2OS cells were seeded in 35 mm confocal dishes for 24 h and transfected with GFP-tagged proteins. After 24 h of transfection, the cells were irradiated with a 405 nm laser (Nikon Eclipse Ti2 and Andor Dragonfly system), and images were recorded for the indicated times. The fluorescence intensity of the laser tracks and the DNA fiber tract lengths were measured using ImageJ.

### *In situ* PLA

PTIP-depleted shBRCA1 HeLa cells were reconstituted with FLAG-PTIP-WT or FLAG-PTIP-4KR mutant. Empty vector was used as negative control. Cells were labeled with EdU (10 μM, 15 min), followed by HU (4 mM, 3 h) treatment. After PBS washing, cells were permeabilized with 0.2% Triton X-100 at RT for 10 min and fixed with 4% paraformaldehyde. Then, cells were washed with PBS three times, and biotin azide is covalently linked to an alkyne functional group on EdU *via* a “Click-it” reaction. Following incubation with primary antibodies, the PLA was performed using the Duolink *In Situ* Red Starter kit (Sigma-Aldrich) following the manufacturer’s instructions. After washing, cells were incubated with DAPI and then imaged on an Olympus microscope equipped with a digital camera. At least 100 cells were counted in each condition.

### Metaphase spread assay

After treatment with or without HU (4 mM) for 5 h, shBRCA1 HeLa cells transfected with siCon, siPTIP, and siPTIP reconstituted with FLAG-PTIP-WT or FLAG-PTIP-4KR were washed with PBS and cultured in fresh medium for 24 h. Cells were harvested after nocodazole (10 μM, 6 h) treatment. The cells were resuspended in warmed 75 mM KCl containing 10% FBS for 10 min at 37 °C and were then fixed with methanol: acetic acid (3:1) at 4 °C overnight. Then, the cells were dropped onto cold slides and air dried overnight. The slides were mounted in Prolong Gold Antifade (Invitrogen) with DAPI before images were captured under a 60 × /1.4 oil immersion objective (Nikon Eclipse Ti2 and Andor fusion software) and analyzed with ImageJ.

### Cell viability assay

Cells were seeded in 6-well plates at a density of 500 cells/well. Then, the cells were treated with the indicated dose of PARPi (AZD2188) and cultured for 10 to 14 days. Colonies were fixed and stained with 0.05% methylene blue including methanol for 20 min at RT. Then, the number of colonies were calculated by GelCount (Oxford Optronix). Experiments repeated three times.

### Statistics

Data were analyzed using GraphPad Prism software according to corresponding statistical tests (student’s *t* test), and the resulting *p* values are indicated in the figure legends. A *p* value < 0.05 was considered statistically significant.

## Data availability

All data generated and analyzed in this study are included in this published article and its supplementary information files.

## Supporting information

This article contains supporting information.

## Conflict of interest

The authors declare no conflict of interest with the contents of this article.
