# Supplementary material for: PTIP UFMylation promotes replication fork degradation in BRCA1-deficient cells
Source: J Biol Chem. 2024 Apr 22;300(6):107312. doi: 10.1016/j.jbc.2024.107312 (PMC11130726; doi:10.1016/j.jbc.2024.107312)
Supplement: Supporting Information [file mmc1.pdf]

S-1

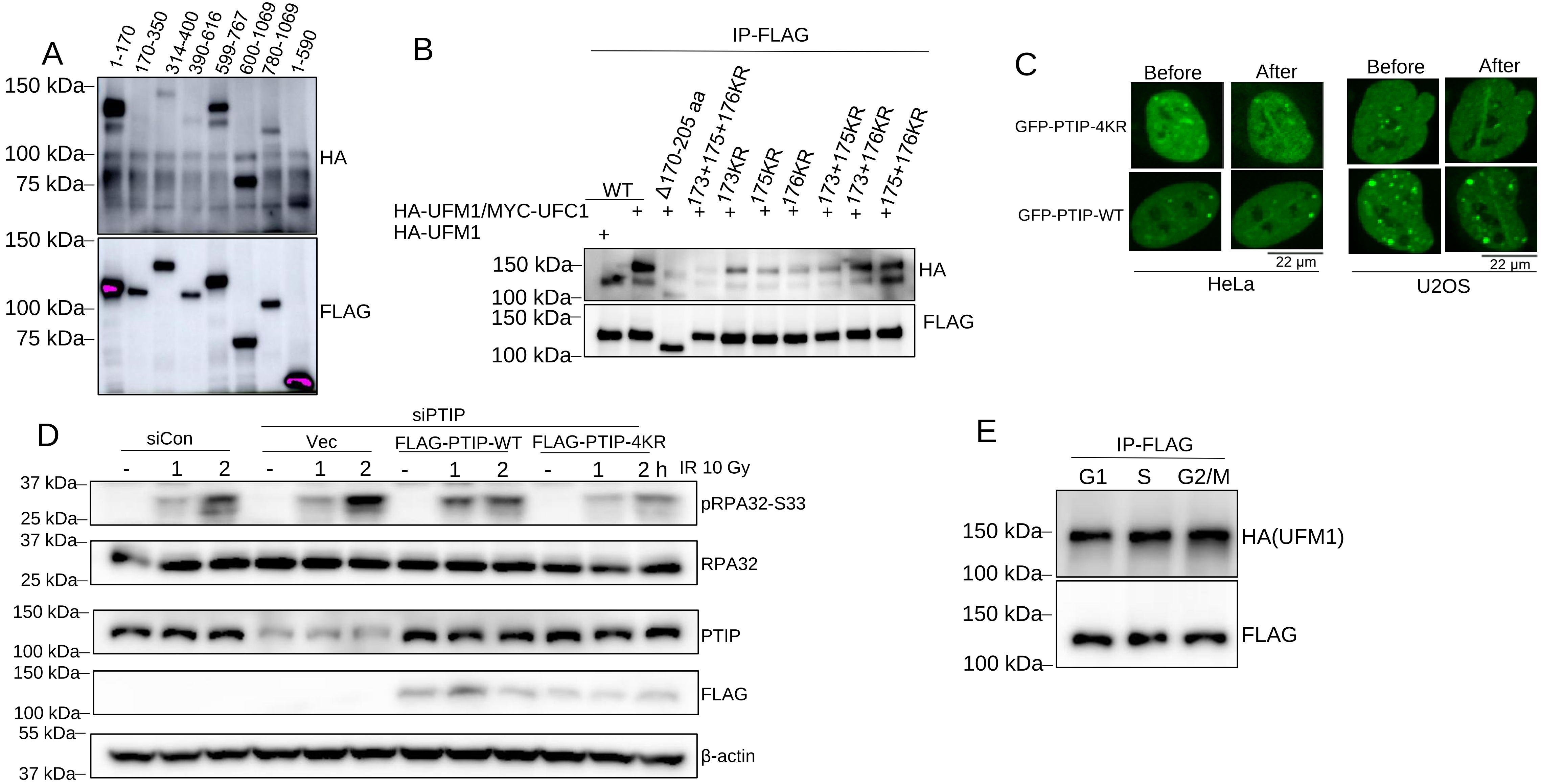

Supplemental Figure 1 PTIP is UFM1-modified in *vivo* in response to replication stress. (A) PTIP fragments, HA- UFM1-ΔC2, and MYC-UFC1 were transfected into HEK293T cells, and UFM1-conjugated PTIP enriched by FLAG-M2 beads was detected by western blotting. (B) Wild type (WT) and PTIP mutants were co-transfected with HA-UFM1-ΔC2 and MYC-UFC1 into HEK293T cells, and UFM1-conjugated PTIP was enriched using FLAG-M2 beads and detected by western blotting. (C) GFP-tagged PTIP-WT or a PTIP-4KR mutant were transfected into HeLa (upper) or U2OS (lower) cells and the cells were micro-irradiated. (D) siControl, siPTIP, and siPTIP reconstituted with FLAG-PTIP-WT or FLAG-PTIP-4KR mutant cells treated with IR (10 Gy) were collected in the indicated timepoints for western blot analysis with indicated antibodies. (E) HeLa cells were transfected with the indicated constructs and synchronized at G1, S or G2/M. UFM1-conjugated PTIP was pulled down by FLAG-M2 beads and analyzed by western blotting.

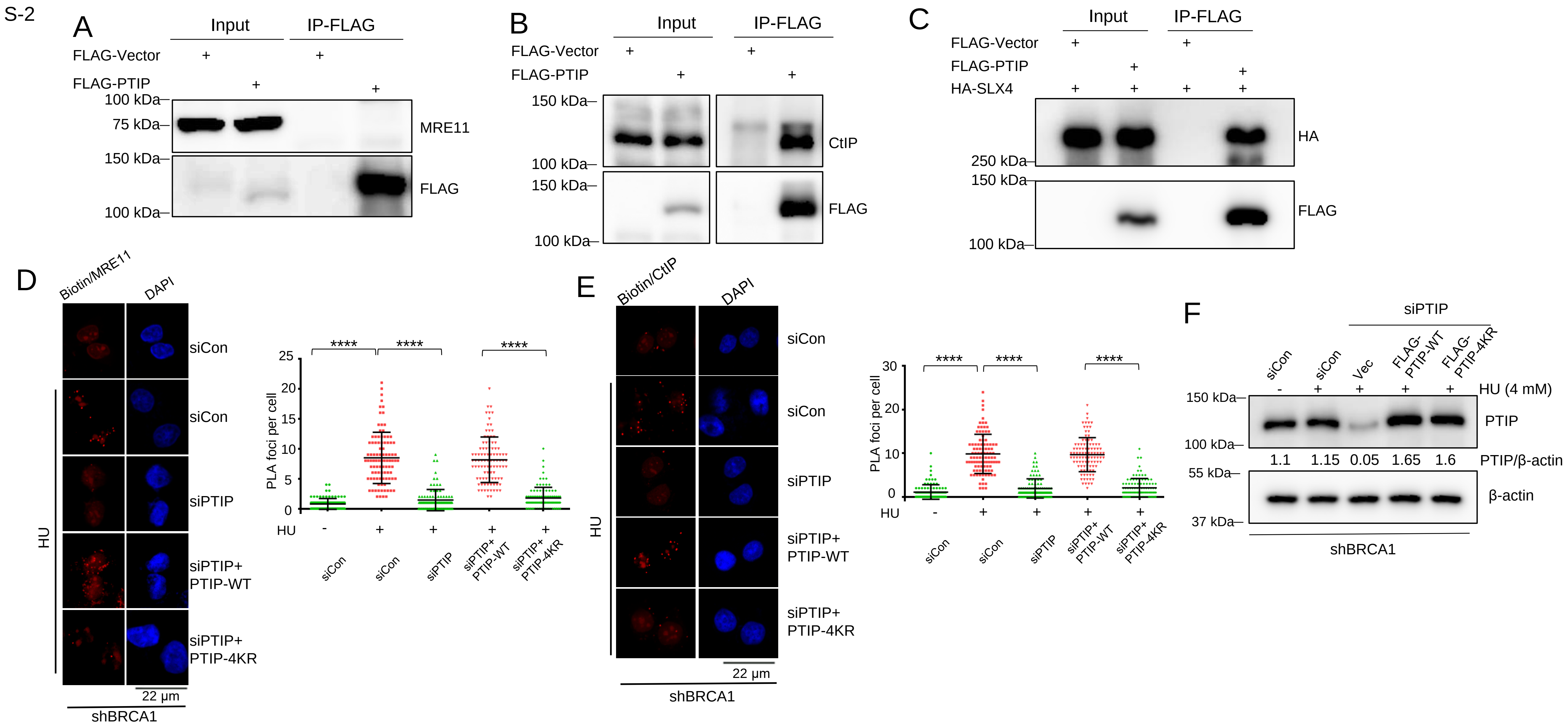

Supplemental Figure 2 UFMylation promotes nascent DNA degradation in BRCA1-deficient cells. (A) FLAG-Vector or FLAG-tagged PTIP were transfected into HEK293T cells for 48 h, and cells were harvested for FLAG-tagged PTIP purification under nature condition followed by immunoblotting analysis with anti-MRE11 or anti-FLAG antibody. (B) HEK293T cells expressing FLAG-Vector or FLAG-PTIP were collected and FLAG-tagged proteins were pulled down with FLAG-M2 beads under nature condition followed by western blotting with anti-CtIP or anti-FLAG antibody. (C) Purified FLAG-tagged proteins from HEK293T cells expressing HA-tagged SLX4 with or without FLAG-PTIP were analyzed by immunoblotting with anti-HA or anti-FLAG antibody. (D, E) Indicated shBRCA1 cells were labeled with 10 μM EdU for 10 min before HU (4 mM, 3 h) treatment. Click chemistry was applied to conjugate biotin to EdU. Representative images of PLA foci obtained in the indicated shBRCA1 cells for MRE11 shown in (D, left) and CtIP shown in (E, left). Quantification of the number of PLA foci per cell was shown in (D, right) or (E, right). P values were derived from a one-way ANOVA with Tukey's multiple comparisons test. (F) Knockdown efficiency of PTIP and expression of PTIP-WT and PTIP-4KR mutant.
